# Liver-Mediated Adaptive Immune Tolerance

**DOI:** 10.3389/fimmu.2019.02525

**Published:** 2019-11-05

**Authors:** Meijuan Zheng, Zhigang Tian

**Affiliations:** ^1^Department of Clinical Laboratory, First Affiliated Hospital of Anhui Medical University, Hefei, China; ^2^Hefei National Laboratory for Physical Sciences at Microscale, CAS Key Laboratory of Innate Immunity and Chronic Disease, Division of Molecular Medicine, School of Life Sciences, University of Science and Technology of China, Hefei, China; ^3^Institute of Immunology, University of Science and Technology of China, Hefei, China

**Keywords:** liver tolerance, T cell dysfunction, innate cell dysfunction, immune regulation, liver-draining lymph node, liver diseases

## Abstract

The liver is an immunologically tolerant organ that is uniquely equipped to limit hypersensitivity to food-derived antigens and bacterial products through the portal vein and can feasibly accept liver allografts. The adaptive immune response is a major branch of the immune system that induces organ/tissue-localized and systematic responses against pathogens and tumors while promoting self-tolerance. Persistent infection of the liver with a virus or other pathogen typically results in tolerance, which is a key feature of the liver. The liver's immunosuppressive microenvironment means that hepatic adaptive immune cells become readily tolerogenic, promoting the death of effector cells and the “education” of regulatory cells. The above mechanisms may result in the clonal deletion, exhaustion, or inhibition of peripheral T cells, which are key players in the adaptive immune response. These tolerance mechanisms are believed to be responsible for almost all liver diseases. However, optimal protective adaptive immune responses may be achieved through checkpoint immunotherapy and the modulation of hepatic innate immune cells in the host. In this review, we focus on the mechanisms involved in hepatic adaptive immune tolerance, the liver diseases caused thereby, and the therapeutic strategies needed to overcome this tolerance.

## Introduction

As the largest organ in the body, the liver has a rich circulatory supply, receiving blood from both the hepatic artery and the portal vein. As a result, the liver comes into contact with a large proportion of microbial products, as well as harmless food-derived antigens, via the intestines. A high level of exposure to these antigens endows the liver with a distinctive form of immune privilege. This so-called immunotolerance ensures that the liver does not mount a strong immune response against gastrointestinal tract-derived molecules and pathogens. This tolerance effect is also evidenced by the fact that the liver readily accepts allografts, despite a major histocompatibility complex (MHC) mismatch, as seen early on in the pig model of transplantation (1).

Later studies have shown that the liver can accept subsequent non-hepatic allografts from the same donor by inducing systemic immune tolerance (2). Similarly, the tolerance induced via the liver-mediated expression of exogenous proteins is used in gene therapy for hemophilia, metabolic disorders, lysosomal storage disorders (3), and even autoimmune diseases (4). However, the hepatic immune tolerogenic environment is also exploited by hepatitis viruses, parasites, and tumors and can lead to persistent infection and rapid cancer progression.

Adaptive immunity plays a key role in defending the host against pathogens and tumors. The liver determines organ/tissue-localized and systematic adaptive immune responses, highlighting the link between adaptive immune responses and the hepatic microenvironment (5). Evidence also suggests that relationships exist between adaptive immune responses and the hepatic tolerogenic microenvironment (6). This tolerogenic microenvironment leads to liver T cell dysfunction, including clonal deletion, anergy, senescence, deviation, and exhaustion. The liver is home to large numbers of hepatocytes, nonparenchymal cells, and lymphocytes (7). This means that complex interactions between these cells contribute to the induction of adaptive immune tolerance in the liver. For example, parenchymal and nonparenchymal cells suppress the adaptive immune response, leading to hepatic T cell dysfunction, partially as a result of inhibitory receptor and anti-inflammatory cytokine expression (8).

Here, we describe hepatic adaptive immune cell-related dysfunction in the context of liver-mediated adaptive immune tolerance. We focused on: (i) T cell dysfunction, including anergy, exhaustion, and apoptosis, (ii) the regulatory mechanisms involved in the induction of T cell dysfunction, (iii) the current understanding of the role of T cell dysfunction in liver disease, and (iv) the therapeutic strategies developed to counteract adaptive immune tolerance, to illustrate the complexity of and challenges related to liver-mediated adaptive immune tolerance.

## The Adaptive Immune Tolerance Mechanisms

How does the liver tolerize adaptive immune cells? Since adaptive immune cells are easier to render tolerant in the liver than in other organs, the liver has been classically referred to as a “graveyard” for effector T cells and a “school” for regulating cells. In this regard, several reports demonstrate that local antigen presentation in the liver results in T cell apoptosis (9, 10).

### The Liver Acts as a T Cell “Graveyard”

The classic hypothesis that the liver functions as a “graveyard” for T cells suggests that the liver represents a specific site for the apoptosis of activated T cells (11) that become trapped and eventually destroyed in the liver by clonal deletion, clonal anergy, clonal deviation, and T cell exhaustion.

Clonal deletion is a process whereby T and B cells expressing antigen-specific receptors with self-reactive specificities are deleted during their development. Huang and colleagues used T cell receptor (TCR) transgenic mice to show that activated T cells could be programmed to undergo apoptosis in the liver through peptide injection (12). Another study suggested that the liver trapped and passively sequestered activated CD8^+^ T cells (13). In line with these findings, another transgenic mouse model indicated that hepatocyte-activated CD8^+^ T cells with increased expression of Bim were associated with premature death (14). A landmark study by the Bertolino group demonstrated that CD8^+^ T cells undergoing emperipolesis were endocytosed and deleted by hepatocytes, suggesting that “suicidal emperipolesis” is a unique mechanism of peripheral deletion (15). Thus, this “suicidal emperipolesis” plays an important role in liver-activated autoreactive CD8^+^ T cell clearance and immune homeostasis in the liver (16).

Clonal anergy refers to a state of inactivation experienced by self-reactive lymphocytes. Anergic lymphocytes cannot induce strong immunity in healthy individuals. Liver sinusoidal endothelial cells (LSECs), acting as antigen-presenting cells, in the absence of accessory signals reportedly induce anergy in T cells within the hepatic microenvironment (17). Another study supports the idea that plasmacytoid dendritic cells (pDCs) may also lead to the inhibition of T cell activity in the liver, resulting in the anergy or deletion of antigen-specific T cells (18).

Clonal deviation is the process whereby naïve CD4^+^ T cells preferentially assume the Th2 but not the Th1 or the Th17 phenotype during differentiation in the liver. The priming of naïve CD4^+^ T cells by liver sinusoidal endothelial cells (LSECs) fails to promote their differentiation into Th1 cells, even with the exogenous administration of the cytokines IL-1β, IL-12, and IL-18 (19). Thus, LSECs suppress the IFN-γ-producing Th1 cells in favor of the IL-4-expressing Th2 cells, contributing to the process of immune T cell deviation in the liver (20).

Another form of T cell dysfunction, T cell exhaustion, is often associated with chronic infection and tumorigenesis (21). An exhausted T cell is characterized by impaired effector functions and proliferative capacity, as well as altered transcriptional, epigenetic, and metabolic signatures, including the overexpression of inhibitory receptors and a dysregulated cytokine milieu (22, 23). The first report of T cell exhaustion occurred in a mouse model of noncytopathic lymphocytic choriomeningitis virus (LCMV) infection, in which exhausted CD8^+^ T cells displayed impaired effector functions compared to functional CD8^+^ T cells (24). This begs the question of what causes T cell exhaustion in the first place.

Firstly, persistently high levels of antigen contribute to T cell exhaustion (25). A threshold of intrahepatic antigen levels tunes the fate of cytotoxic T lymphocyte (CTL) function, and high levels of antigen maintain an exhausted T cell phenotype (26). Secondly, altered inflammatory and tissue microenvironments play an important role in inducing T cell tolerance (22). In such circumstances, T cells lose their robust effector functions, accompanied by an increase in the expression of multiple inhibitory receptors, such as PD-1, CTLA-4, LAG-3, and TIM-3.

In addition, T cells receive inhibitory signals from various immunosuppressive cytokines. The phenomenon of T cell exhaustion has been reported both in chronic infections and cancer of the liver. Exhausted hepatic T cells are closely related to inefficient clearance of persisting pathogens and tumorigenesis in chronic liver diseases, including hepatitis B and C, malaria, schistosomiasis, and liver cancers. Thus, T cell exhaustion is considered to be associated with hepatic tolerogenic characteristics in liver diseases. Recently, the signal-regulatory protein α was shown to act as an inhibitory receptor when expressed on CD8^+^ T cells during chronic exhaustion in chronic hepatitis C virus (HCV) infection (27).

### The Liver Acts as a School to Educate T Cells

The coordination between innate and adaptive immune cells often occurs when confronting liver disease, as the unique structure of this organ facilitates interactions between these cells. There are several hepatic antigen presenting cells (APCs) including resident hepatocytes and non-parenchymal cells like DCs, LSECs, Kupffer cells, and hepatic stellate cells (HSCs) involved in antigen presentation, which facilitate adaptive immune tolerance in the liver (28). During the induction of liver immune tolerance, cytokines like IL-10, TGF-β, and IFN-γ are thought to be involved in the development of chronic liver disease and T cell dysfunction (29–31). In the liver environment, multiple factors, including APCs, the site of primary T cell activation, and altered inflammation, dictate the immune outcomes of intrahepatic T cells (32, 33).

Hepatocytes, which do not normally express MHC class II molecules, acquire the ability to express MHC II and activate CD4^+^ T cells during hepatitis (34). Under specific circumstances, however, antigen presentation by hepatocytes can promote immune tolerance. For instance, MHC II-expressing hepatocytes seem to be associated with defective CD4^+^ and CD8^+^ T cell function and higher LCMV titers in class II transactivator molecule (CIITA)-transgenic mice compared with nontransgenic mice (35). Furthermore, the adeno-associated viral vector-mediated expression of a single MHC I allele in hepatocytes induced tolerance toward an allogeneic graft in a transfer experiment involving liver-generated CD8^+^ regulatory T cells (Tregs) (36).

A recent study showed that Qa-1 expression in hepatocytes with NKG2A^+^ natural killer (NK) cells induced CD8^+^ T cell exhaustion and persistent HCV infection in humanized C/O^Tg^ mice (37). Interestingly, hepatocytes are also capable of converting CD4^+^ T cells into Foxp3^+^ Tregs *in vitro*, resulting in the Treg-mediated suppression of the CD4^+^ T cell response via the Notch signaling pathway (38). Together, these observations indicate that hepatocytes mediate T cell dysfunction in the liver.

The DCs are professional antigen-presenting cells (APCs) that migrate to the draining lymph node and present antigens to T cells (39, 40). Hepatic DCs exhibit an immature phenotype, thus maintaining liver tolerance (41). More importantly, tolerogenic DCs, associated with low MHC class I and II levels and a high expression of T cell coinhibitory ligands, mediate tolerogenic effects, including T cell deletion, anergy, Th2 polarization, and the induction of Tregs (42). Tolerogenic DCs also show considerable promise in the control of autoimmune diseases and allograft rejection (43, 44) by promoting tolerance within the hepatic microenvironment. Liver DCs secrete IL-10 and are associated with reduced T cell proliferation and function compared to blood DCs (45).

Kupffer cells account for the largest population of macrophages in the liver. Under many circumstances, Kupffer cells play an important role in antigen uptake and pathogen clearance. However, during homeostasis, Kupffer cells secrete anti-inflammatory soluble factors, such as IL-10, to maintain hepatic tolerance (46, 47). In addition, Kupffer cells reportedly mediate T cell suppression, without the need for cytokines like IL-10, TGF-β, and nitric oxide (48).

The hepatic stellate cells (HSCs) and LSECs are well-characterized liver-resident APCs that are capable of tolerizing T cells. For example, TGF-β1 produced by HSCs inhibits T cells via glycoprotein A repetitions predominant (GARP)-dependent expression on HSCs (49). Moreover, the expression of B7-H1 on HSCs contributes to the regulation of T cell responses by promoting their apoptosis (50). Within the hepatic microenvironment, LSECs can also tolerize both CD4^+^ and CD8^+^ T cells. Furthermore, while LSECs can prime CD4^+^ T cells, these CD4^+^ T cells do not acquire a Th1 phenotype (19). Antigen cross-presentation by LSECs to CD8^+^ T cells also leads to tolerance rather than CD8^+^ T cell activation (51).

The NK cells, which belong to a major group of innate immune cells in the liver, contribute to host defense against virally infected cells and tumors. Mice reportedly contain two liver NK cell subsets, which are referred to as conventional NK cells (which enter the circulation) and tissue-resident NK cells (52, 53). The markers CD49a and DX5 can be used to subdivide murine NK cells into conventional (CD49a^+^DX5-) and liver-resident (CD49a-DX5^+^) NK cells (54). Similarly, human livers are also populated with two overlapping NK cell subsets (55).

Generally, NK cell function is controlled by a diverse set of activating and inhibitory receptors, the balance between which also contributes to the regulation of T cells (56, 57). For example, hepatic conventional NK cells contribute to effective anti-hepatitis B virus (HBV) T cell responses, while liver-resident NK cells directly suppress T cell responses through the programmed cell death-1 ligand-receptor (PDL1-PD1) axis (58, 59). Impaired NK cell function is accompanied by weakened cytotoxic CD8^+^ T cell activity in persistent viral infections (60). Indirectly, NK cells also diminish CD8^+^ T cell responses during chronic infection by interacting with DCs (61).

Interestingly, the hepatic NK cell-associated modulation of the effector T cell response is, in turn, regulated by the liver microenvironment, such as the presence of IL-10 (62). In addition, HBV-specific CD8^+^ T cells become susceptible to TNF-related apoptosis-inducing ligand (TRAIL)-expressing NK cell-mediated killing by upregulated TRAIL-R2 expression in patients with chronic HBV infection (CHB), indicating that NK cells downregulate HBV-specific CD8^+^ T cell responses (63, 64). In this scenario, upon TRAIL and NKG2D blockade, NK cell-mediated HBV-specific T cell function is also enhanced in patients with CHB who are treated with a nucleos(t)ide analog (65).

Also residing in the liver are natural killer T (NKT) cells, innate-like T cells that modulate the hepatic immune response by producing pro- and anti-inflammatory cytokines upon activation. There are two types of NKT cells, type I and type II NKT cells. Type I NKT cells express a semi-invariant TCR and is also referred to as invariant (i) NKT cells. By contrast, type II NKT cells express a relatively diverse TCR repertoire. Type II NKT cells conversely appear to be more abundant than type I NKT cells in humans, but in liver diseases, they are similar to type I NKT cells in phenotype and function (66).

By bridging the innate and adaptive responses, NKT cells act as immunoregulators during immunological liver disease. Lan et al. (67) revealed that the pyroptosis of iNKT cells through OX40 signaling can lead to liver inflammation and damage, suggesting that NKT cells play an important role in liver homeostasis. On the other hand, activated NKT cells contribute to the recruitment of Tregs via the CXCR3-CXCL10 pathway (68). The NKT cells also reportedly promote the priming of IL-10-producing CD8^+^ T cells by hepatocytes in order to limit liver injury (69).

Similar to NKT cells, mucosal-associated invariant T (MAIT) cells are the T cell subpopulation restricted to the MHC-I-related (MRI) molecule MRI populated in humans that produces a Th1 and Th17 cytokine milieu (70). The presence of highly enriched MAIT cells in the human liver suggests the importance of these innate cells in the control of liver infections (71, 72). However, in patients with chronic HCV infections, CD8^+^CD161^++^TCRVa7.2^+^ MAIT cells exhibit exhausted features, thereby contributing to HCV persistence (73). In line with these findings, MAIT cells from patients with chronic hepatitis delta virus (HDV) are functionally impaired and subsequently lost during HDV infection (74). In patients with hepatocellular carcinoma (HCC), tumor-educated MAIT cells upregulate inhibitory receptors and display functional impairment, both of which correlate with HCC progression (75).

The Tregs negatively regulate effective T cell immune responses via the production of immunosuppressive cytokines (including IL-10 and TGF-β) during chronic infection and are considered to be a potential target for the treatment of patients with CHB (7). Through upregulated Tregs, IL-33 exerts a negative effect on CD4^+^ T cell proliferation and alleviates hepatitis (76). Similarly, it was found that Tregs orchestrate CD8^+^ T cell exhaustion by engaging the PD-1 inhibitory pathway during LCMV infection (77). However, circulating CD4^+^CD25^+^ regulatory T cells exist in patients with resolved HBV infection (78). Furthermore, the numbers of these regulatory cells are increased and correlate with hepatic inflammation in patients with hepatitis B (79). Therefore, Tregs might play a role in anti-inflammatory activity and need to be more thoroughly assessed (80).

In contrast to T cell tolerance, antibody response to HBV proteins does not provide evidence for B cell tolerance during HBV infection. For example, antibodies specific to the HBV core antigen (anti-HBc) are clearly detectable during acute HBV infection (81). Interestingly, anti-HBc antibodies can be elicited in patients with CHB and are more abundant in CHB infection compared with in patients with self-limited infections (82, 83). Furthermore, highly active B cell responses are indicated during chronic HBV infection through gene expression profiling (84). In contrast, hepatitis B surface antibodies (anti-HBs) are considered to be protective and are commonly associated with viral control and the resolution of clinical disease. A recent study demonstrated that HBcAg-specific B cells and HBsAg-specific B cells were different in phenotype and function but shared an increased mRNA expression of genes linked with the role of cross-presentation and innate immunity in patients with CHB (85). Overall, the above results indicate that HBV-specific humoral responses are apparently not suppressed in the liver.

### The Role of Liver-Draining Lymph Nodes (LNs) in the Induction of Hepatic Immune Tolerance

Although the “graveyard” and “school” models are adequate under certain circumstances, some argue that T cell tolerance is not the direct consequence of local antigen-presentation (86), and that the “graveyard” theory cannot account for the existence of efficient immune responses under different conditions (87). Since many other factors are involved, the two models may not present full explanations of the tolerogenic mechanisms at play in the liver, and additional hypotheses may be required.

The liver produces considerable amounts of lymphatic fluid, which is one of the two major sources of abdominal lymph. Hepatic lymph is thought to originate from the filtration of the sinusoids into the space of Disse, even before the lymph drains from the liver through the lymphatic vessels to the draining LN (88). Although the liver-draining LNs are well-reported in humans, the portal and celiac liver-draining LNs in the mouse have only recently been clearly described in studies that used Evans blue dye or infection with an adenovirus vector carrying the enhanced green fluorescent protein gene (Ad-EGFP) to track hepatic lymphatic draining (89, 90). These studies also show that DCs exit the liver and migrate to the liver-draining LNs, where they prime and facilitate specific T cell responses.

Interestingly, portal and celiac LNs appear to be independent liver-draining LNs, with different cellular compositions and modes of organogenesis. Furthermore, the portal LN participates in oral tolerance via Treg induction, while the celiac LN facilitates effective T cell responses (91). The immune response that occurs in liver-draining LNs is associated with the liver microenvironment, which is considerably different from that of the spleen. Importantly, liver-draining LNs are implicated in chronic human disease (92, 93). Recent progress in research studies related to the association between the liver and human portal LNs indicates that a paucity of DCs in human portal LNs contributes to hepatic immune tolerance (94). In addition, the regional immunity implicated in liver homeostasis and disease is associated with tissue-specific immune cell subsets and their interactions with the liver (7). Thus, a major aspect of liver function is dependent on specific hepatic immune cell subsets, which may, in turn, be influenced by the immune responses modulated by liver-draining LNs.

Moreover, studies indicate that liver inflammation is also involved in liver tolerance. Patients with chronic hepatitis B have fewer signs of inflammation than those with acute hepatitis B who clear the viral infection and display significant inflammation (95). Furthermore, circulating monocytes under inflammatory stimuli can activate autologous HBV-specific T cells during chronic HBV infection, suggesting that inflammatory conditions might have an impact on intrahepatic HBV-specific T cells (80, 96).

In a study conducted in chimpanzees with chronic infections, agonists of toll-like receptor (TLR) 7 activated TLR-7 signaling and reversed immune tolerance associated with significant intrahepatic inflammation (97). Similarly, TLR 7 agonists appear to enhance T cell and NK cell activities in patients with CHB who are subjected to nucleos(t)ide therapy (98). The above results, taken together, support the hypothesis that inflammatory events in the liver might alter the features of liver tolerance. However, liver tolerance is not absolute during viral hepatitis infection. For example, patients with acute hepatitis elicit an effective adaptive immune response but lack immune tolerance to hepatitis A, B, and C (99–101).

**Figure 1:**
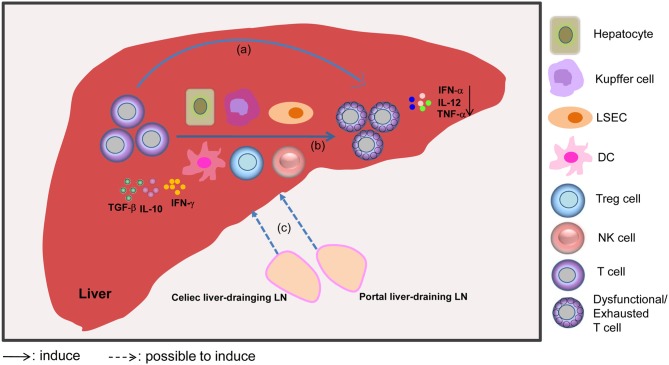
Mechanisms involved in liver-mediated adaptive immune tolerance. The diagram outlines that the liver acts as a “graveyard” or killing field for activated T cells, leading to the apoptosis of activated T cells through clonal deletion, clonal anergy, clonal deviation, or exhaustion (a). Alternatively, the liver can act as a school to educate T cells, which means that T cells can be subjected to regulation by liver APCs, including dendritic cells (DCs), hepatocytes, Kupffer cells, liver sinusoidal endothelial cells (LSECs), regulatory T cells (Tregs), and NK cells, and cytokines like IL-10, TGF-β, and IFN-γ, which promote T cell dysfunction (b). Furthermore, dysfunctional T cells may be induced by the liver-draining LN environment through antigen presentation. The liver-draining portal and celiac lymph nodes (LNs) play an important role in regulating hepatic immune tolerance (c). Moreover, mild or absent signs of liver inflammation, as evidenced by reduced levels of IFN-α, IL-12, and TNF-α cytokines, are also associated with the scenario of liver tolerance.

In summary, several mechanisms are involved in the induction of T cell dysfunction in the liver. On the one hand, the liver is seen as a “graveyard” or killing field for activated T cells, because it can induce T cell dysfunction in the local microenvironment. On the other hand, the large population of liver APCs, and cytokines like IL-10, TGF-β, and IFN-γ lead to the negative regulation and further dysfunction of T cells. Additionally, the celiac and portal liver-draining LNs apparently play key roles in promoting liver-mediated adaptive immune tolerance through the induction of Tregs and paucity of DCs. Moreover, a lack of inflammatory events under certain circumstances is also associated with T cell dysfunction (Figure 1).

## Adaptive Immune Tolerance in Liver Disease

Under certain pathological circumstances, pathogens, including HBV, HCV, malaria, and schistosomes, exploit the liver's tolerogenic mechanisms to establish persistent infections. For the same reason, the hepatic immunotolerant microenvironment further facilitates the progression of chronic infection to liver fibrosis, cirrhosis, and cancer. Based on the mechanisms involved in liver tolerance, the presence of dysfunctional adaptive immune cells and immunosuppressive regulatory cells is a hallmark of chronic liver disease, including chronic infections and HCC.

### Chronic Liver Infection

Effective T cell responses mediate viral clearance in murine models of HBV infection (58). Intrahepatic HBV-specific CD8^+^ T cells contribute to viral elimination and disease pathogenesis in chimpanzees acutely infected with HBV (102). Similarly, patients with acute HBV infection reportedly have enhanced HBV-specific CD8^+^ T cell responses, which are associated with viral control (103, 104).

Conversely, patients with chronic HBV exhibit HBV-specific CD8^+^ T cell dysfunction, with increased frequencies and intensities of PD-1 expression (105). These findings were reported in the first study to show that HBV-specific CD8^+^ T cells in humans can be exhausted. A recent study using peptide-loaded MHC I tetramers suggests that the phenotypic and functional differences of HBV-specific CD8^+^ T cells can be detected by targeting core vs. polymerase antigen epitopes in patients with CHB, indicating that the molecular mechanisms underlying dysfunctional CD8^+^ T cell populations are not homogeneous in patients with CHB patients (106).

Intrahepatic HCV-specific CD8^+^ T cells have an impaired ability to produce IFN-γ, resulting in a failure to control HCV infection in patients in whom the infection is chronic (107). In addition, it is found that HBV clearance can be achieved by the reconstitution of HBV-specific CD8^+^ T cells, thereby reestablishing adaptive immune responses and reversing HBV-specific tolerance (108). The upregulation of inhibitory receptors on T cells in chronic infection is indicative of T cell exhaustion during viral persistence. For instance, T cell dysfunction is associated with the increased expression of PD-1 and CTLA-4 in patients with CHB compared with in healthy controls (109). Furthermore, in chronic HCV infection, HCV-specific CD8^+^ T cell exhaustion is associated with high expression of inhibitory receptors, while the population of PD-1^−^TIM-3^−^HCV-specific CD8^+^ T cells outnumbers the frequency of PD-1^+^TIM-3^+^T cells in acute resolving HCV infection (110).

Regarding parasitic infections, malaria, and schistosomiasis also establish pathogen persistence and liver tolerance. The CD8^+^ T cells generated in the liver fail to eliminate malaria-causing sporozoites owing to hepatic immune tolerance (111). Moreover, the poor effector functions of exhausted parasite-specific T cells during malaria infection are also linked to PD-1 expression (112). During hepatic schistosomiasis, Th2 cells and Tregs dominate the immune response and release immunosuppressive cytokines, including IL-10 and TGF-β in the liver (113, 114). In addition, many other factors, including the ligands of inhibitory receptors expressed on APCs, account for the failure of dysfunctional T cells to eliminate pathogenic infections in the liver. For example, owing to the selective overexpression of PD-L1 on the surface of macrophages, both CD4^+^ T and CD8^+^ T cells become anergized by the *Schistosoma mansoni* parasite (115).

### Liver Cancer

Antigen-specific T cells play a key role in controlling cancer, but similar to chronic viral infections, persistent tumor cell stimulation causes T cell exhaustion (25). A single T cell database revealed that exhausted tumor-infiltrating CD8^+^ T cells preferentially accumulate in the HCC tumor microenvironment (116). In addition, the epigenetic profile of exhausted T cells is distinct from that of functional effector and memory T cells (117). In the context of the tumor microenvironment, exhausted CD8^+^ T cells exhibit reduced effector functions and proliferative capacity. Furthermore, in HCC tissue, CD4^+^ and CD8^+^ T cells display increased expression of inhibitory receptors such as PD-1, TIM-3, LAG-3, and CTLA-4 (118).

Moreover, HCC specimens reportedly harbor exhausted CD8^+^ T cells with varying levels of PD-1 expression. The PD-1^High^ CD8^+^ T cell subset co-expresses high levels of TIM-3 and LAG-3, as is characterized by low IFN-γ and TNF production, indicating that the expression of PD-1 on CD8^+^ T cells arises as a result of the HCC microenvironment (119). A previous study has shown that the upregulation of Lnc-TIM-3, which specifically binds to TIM-3, can result in CD8^+^ T cell exhaustion in HCC (120). During chronic liver diseases, CD8^+^ T cells with upregulated TIM-3 expression contribute to CD8^+^ T cell exhaustion. The membrane-bound TIM-3 can be cleaved from the cell membrane and yield serum soluble TIM-3, which is associated with liver dysfunction in patients with HCC (121).

Professional or conventional APCs, which can negatively affect T cell function, also play important roles in the regulation of the immune response. Recently, myeloid (m)DCs were found to be functionally impaired in patients with HCC (122), while PD-1 expression on mDCs contributed to the inhibition of CD8^+^ T cell function (123). Kupffer cells also mediate the suppression of CD8^+^ T cells in human HCC, via the B7-H1/PD-1 axis, whereby tumor-associated IL-10 production contributes to the increased B7-H1 expression on Kupffer cells (124).

An important subset of innate immune cells, dysfunctional NK cells are also associated with tumor development (125) and are implicated in the development of HCC. For example, the high expression of NKG2A on NK cells contributes to NK cell exhaustion, which correlates with a poor prognosis for patients with HCC (126). Similarly to NKG2A^+^ NK cells, the HCC microenvironment harbors high numbers of functionally exhausted CD96^+^ NK cells and a few functionally active CD160^+^ NK cells in patients with HCC (127, 128).

Liver-infiltrating CD11b^−^CD27^−^NK cells represent another dysfunctional subset, closely associated with HCC progression (129). In line with the above findings, dysfunctional DCs, Kupffer cells, and NK cells are associated with T cell dysfunction in the HCC microenvironment. Further study is required to delineate the molecular mechanisms involved in the induction of T cell dysfunction, since the heterogeneity of various innate immune cell phenotypes and functions have been well-described.

## Strategies for Reversing T Cell Dysfunction in Liver Disease

In the liver, T cell-mediated immune tolerance is associated with chronic liver disease. Therefore, reversing immunotolerance is thought to be an effective strategy for restoring effective T cell function, and several approaches have been proposed. For example, novel T cell-based vaccines counteract T cell anergy and restore normal CD8^+^ T cell function, contributing to therapeutic immunity in chronic infection (130). A promising report showed that human redirected T cells with HBV-specific TCR can induce antiviral effects in HBV-infected human liver chimeric mice (131). Furthermore, TCR-redirected T cells exhibited the potential for functional degranulation and reduced HBsAg levels in a patient with HBV-related HCC (132).

Interestingly, clinical evidence supports the theory that leukemia recipients with HBV infection undergoing bone marrow transplantation can be cured of functional HBV after bone marrow transfer from naturally HBV-immune or actively immunized donors (133, 134). Using IL-12-based vaccination to counteract liver-induced immunotolerance is also an effective strategy for eliciting robust HBV-specific T cell immunity in an HBV-carrier mouse model (135). Moreover, the blockade of inhibitory signaling pathways to reinvigorate exhausted T cell immune responses is thought to be a promising therapeutic strategy, with the blockade of PD-1 signaling proving the most effective to date in the context of HBV infection (136). Notably, IL-12, as the third signal cytokine, enhances the ability of PD-1 signaling blockade to promote the recovery of functional HBV-specific CD8^+^T cells in patients with chronic HBV (137). The addition of CTLA-4 blocking antibodies can partially lead to the rescue of the effective HBV-specific CD8^+^T cell response in patients with persistent HBV infection (138).

The year 2013 marked a major breakthrough for cancer immunotherapy (139). Among effective cancer immunotherapies, blockage of the checkpoint inhibitors, CTLA-4 and PD-1, has shown the most promise, with many HCC patients increasingly benefiting from more treatment options and combinatorial immune checkpoint inhibitor blockade (140). For instance, blocking NKG2A potentiates tumor-infiltrating CD8^+^ T cell immunity but not NK cells (141). However, this immunosuppressive strategy is hindered by some immunological obstacles, thus resulting in only a minority of tumor patients achieving durable immune responses. Moreover, under certain conditions, CD8^+^ T cell exhaustion may occur in the absence of PD-1 upregulation (142). Therefore, other viable strategies for reversing T cell dysfunction are required to supplement immunotherapy in the context of liver disease.

Several types of parenchymal and nonparenchymal cells also exhibit immunomodulatory functions through their association with T cells in the liver. Therefore, the targeting of immune regulation between APCs or innate immune cells and dysfunctional T cells is expected to have a positive effect on the treatment of liver disease. Evidence suggests that the impairment of DC function is associated with exhausted T cell responses and that CD40-mediated mDC activation rescues intrahepatic anti-HBV CD8^+^ T cells from PD-1-mediated exhaustion (143).

In patients with HCC, both the peripheral and blood DCs co-express PD-1, while the intratumoral transfer of PD-1-deficient DCs elicits tumor-specific CD8^+^ T cell immune responses and restricts tumor growth (123). In chronic HBV infection, HBV-induced monocytes educate NK cells to produce IL-10 via the PDL1/PD-1 pathway, which then contributes to autologous CD4^+^ and CD8^+^ T cell inhibition (144). This suggests that NK cells could be targeted for CHB therapy. Furthermore, the blockade of the checkpoint receptor, TIGIT, promotes NK cell-based tumor-specific T cell immunity, further highlighting the contribution of NK cells to the restoration of tumor-specific CD8^+^ T cell immune responses (145). In particular, the blockade of the inhibitory receptor NKG2A increases NK cell effector function and the associated anti-viral and anti-tumor immunity in chronic liver diseases, such as CHB infection and HCC (126, 146).

Interestingly, the same anti-NKG2A blocking mAb was recently reported to enhance anti-tumor immune responses by unleashing both NK and T cell effector functions (147). In patients with CHC, TRAF1^low^HCV-specific CD8^+^ T cell function is restored through IL-7 plus 4-1BBL and PD-1 blockade treatment, indicating a promising immunotherapy for patients with CHC (148).

## Concluding Remarks

The liver has developed various mechanisms for the induction and maintenance of immune tolerance. Hepatic immunotolerance is associated with the presence of dysfunctional T cells, and the processes of clonal deletion, anergy and exhaustion, dysfunctional regulatory cells, and altered liver inflammatory processes. During chronic liver disease, this tolerogenic state prevents the mounting of an effective adaptive immune cell response against pathogens or tumor cells.

In addition to the immunotherapeutic strategies employed to overcome tolerance in liver disease, several approaches have been developed to reverse T cell dysfunction. For instance, Knolle and colleagues found that even in chronic viral infection, antigen-activated intrahepatic CD8^+^ T cell proliferation was induced by intrahepatic myeloid-cell aggregates for T cell expansion (iMATEs) without causing liver immune pathology via the TLR pathway (149). Although it has not been determined whether iMATEs have a similar structure to that of tertiary lymphoid tissue in local presentation and priming of CD8^+^ T cells, such findings may provide a new way to break T cell tolerance and induce effective anti-pathogen immune responses.

**Figure 2:**
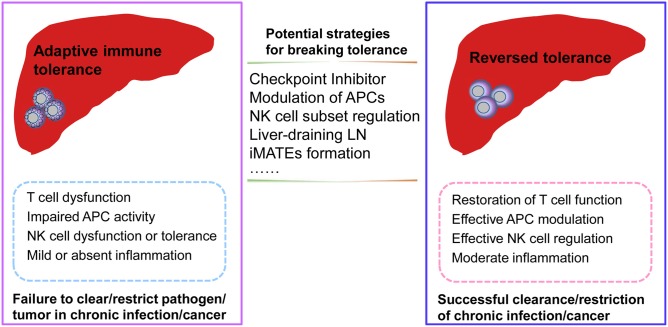
Potential strategies for reversing adaptive immune tolerance in chronic infection or cancer of the liver. During chronic pathogenic infection or tumorigenesis in the liver, dysfunctional adaptive immune responses may be associated with dysfunctional antigen-presenting cells (APCs), natural killer (NK) cell subsets, or T cells. Moreover, mild or absent inflammation may also result in a failure to clear/restrict pathogenic infection or tumor formation. Potential strategies for reversing adaptive tolerance might include checkpoint inhibitor blockade, modulation of specific immune subsets, intrahepatic myeloid-cell aggregates for T cell expansion (iMATES) formation, or liver-draining lymph nodes (LNs) to shape antigen presentation. As a result of these interventions, the restoration of effective immune responses may help to clear or restrict pathogenic infections or tumors with effective T cell function, efficient regulation by specific APC or NK cell subsets, and moderate liver inflammation.

Furthermore, as secondary lymphoid organs, the liver-draining LNs help to shape immune responses in the liver and may play a role in reversing T cell dysfunction by modulating antigen presentation. Moreover, liver-resident NK cell subsets also inhibit T cell function via the PD-L1/PD-1 pathway, while the blockade of PD-L1 abrogates the suppression of T cell function (59). Recent advances in the field of innate immune cell biology, focusing on specific innate immune cell subsets and their different phenotypes and functions, will likely further clarify the regulatory mechanisms and molecular regulators needed to break liver-mediated immune tolerance and reverse adaptive immune cell dysfunction in liver disease. The questions of where and how hepatic immune subsets interact to generate dysfunctional T cells in the context of hepatic immunotolerance remain to be addressed. In summary, additional research is required to identify the innate immune subsets that are involved in inducing T cell dysfunction, the site of their interaction with T cells to render them dysfunctional, and the specific molecular mechanisms that are involved in this complex process (Figure 2).

## Author Contributions

ZT devised this manuscript. MZ wrote this manuscript. ZT and MZ revised the manuscript.

### Conflict of Interest

The authors declare that the research was conducted in the absence of any commercial or financial relationships that could be construed as a potential conflict of interest.

## Abbreviations

TCR: T cell receptor
LSECs: Liver sinusoidal endothelial cells
pDCs: Plasmacytoid dendritic cells
LCMV: Lymphocytic choriomeningitis virus
HCV: Hepatitis C virus
APCs: Antigen presenting cells
HSCs: Hepatic stellate cells
MHC: Major histocompatibility complex
Tregs: Regulatory T cells
NKT: Natural killer T
HBV: Hepatitis B virus
LNs: Lymph nodes
HCC: Hepatocellular carcinoma
(m)DCs: Myeloid
iMATEs: Intrahepatic myeloid-cell aggregates for T cell expansion.
